# Association Between Iron Deficiency Without Anemia and Cognitive Impairment in Children

**DOI:** 10.7759/cureus.89049

**Published:** 2025-07-30

**Authors:** Alka Kumari, Lawson O Obazenu, Manahil Monis, Halimah T Mustapha-Adebiyi, Abali Wandala, Thithiksha Venkata Harischandra, Person Domshak Ayuba, Mahek Thorani, Muhammad Rahim Arshad, Meera Al Shamsi, Alzahrani Renad Mohammed A.

**Affiliations:** 1 Medicine, Liaquat University of Medical and Health Sciences, Hyderabad, PAK; 2 Medicine, University of Ibadan, Ibadan, NGA; 3 Internal Medicine, Neurowave Research Center, Islamabad, PAK; 4 Internal Medicine, Foundation University Medical College, Islamabad, PAK; 5 Internal Medicine, Lagos State University College of Medicine, Lagos, NGA; 6 FCS-Medicine, Universidad Adventista del Plata, Libertador San Martín, ARG; 7 Internal Medicine, Stanley Medical College, Chennai, IND; 8 Family Medicine, University of Jos, Jos, NGA; 9 Internal Medicine, People’s University of Medical and Health Sciences for Women, Karachi, PAK; 10 Internal Medicine, Adam University, Bishkek, KGZ; 11 Genetics, Zayed Higher Organization for People of Determination, Abu Dhabi, ARE; 12 Internal Medicine, Al Baha University, Al Baha, SAU

**Keywords:** behavioral problems, children, cognitive function, iron deficiency, nonanemic iron deficiency

## Abstract

Background: Iron deficiency without anemia is a potentially serious, under-recognized nutritional problem that may adversely influence children's cognitive and behavioral development. Although the connection is supported globally, there is a lack of localized studies in Pakistan. This research investigates the relationship between iron deficiency without anemia and cognitive-behavioral problems in Pakistani children aged 5-18.

Methodology: This cross-sectional study was conducted from February to June 2025, using convenience sampling of schools and communities in Islamabad. A total of 385 children participated. A three-part structured questionnaire, an Iron Deficiency Risk Questionnaire (IDRQ), and the Strengths and Difficulties Questionnaire (SDQ) were used to gather demographic data. Statistical analyses were performed using SPSS Version 26 (IBM Corp., Armonk, NY). They consisted of descriptive statistics, t-tests, analysis of variance (ANOVA), Pearson correlation, and linear regression to evaluate associations between the risk of iron deficiency and cognitive-behavioral functioning.

Results: The sample consisted of 385 participants (*N* = 385), comprising females (*n* = 199, 52%) and males (*n *= 186, 48%), including both urban and rural children. The results of the correlation between the scores on the IDRQ and the SDQ showed a weak positive association (*r* = 0.272, *P* < 0.001). While the correlation was statistically significant, it indicates a modest relationship, suggesting that higher levels of iron deficiency risk are weakly associated with greater cognitive-behavioral difficulties. Children with celiac disease and recurring infections recorded the highest scores. Older children (17-18 years) displayed elevated scores on risk and difficulty compared to younger children (5-7 years). Linear regression analysis revealed that SDQ scores were significantly predicted by IDRQ scores (*B* = 0.669, *P* < 0.001), thereby validating the association.

Conclusions: The results indicate that iron deficiency without anemia is weakly associated with cognitive and behavioral issues among children. Although the correlation is statistically significant, it is modest, indicating the need for further research to fully understand the extent of this relationship. Community-based nutritional interventions are critical in reducing this hidden burden by recognizing that early detection enables better developmental outcomes among Pakistani children.

## Introduction

Iron deficiency is the most common nutritional condition in early childhood and infancy, a time of high iron needs and susceptibility to the development of irreversible neurodevelopmental and cognitive disabilities [1]. Iron deficiency without anemia is a common condition characterized by low ferritin and transferrin saturation levels, despite normal hemoglobin concentration. It often presents with non-specific symptoms, and while oral iron supplements are generally effective, intravenous iron may be necessary for patients who do not respond to oral treatment [2]. Iron deficiency is a major contributor to disease burden in the world, is morbid in children, women, and low-income groups, and impairment can also happen without anemia [3]. Iron deficiency without anemia was prevalent in 57.5% of females and 7.6% of males aged 18-50, with 14% of females progressing to anemia over five years. Similarly, in the United States, iron deficiency affected 9% of toddlers and 9% to 11% of adolescent girls and women, with higher rates in minority, low-income, and multiparous women [4,5]. Although oral iron is considered the first-line therapy, current parenteral preparations offer viable alternatives, particularly for individuals with inflammatory diseases or reduced iron absorption [2].

Severe iron deficit in young children in low-income countries is associated with substantial cognitive and motor impairments, which are, however, complex to prevent. Early interventions and robust delivery systems are crucial ways to enhance iron status and reduce the prevalence of anemia [6]. Iron deficiency affects almost 2 billion people worldwide and is promoted by the poor bioavailability of iron in plant-based diets. It has significant health and economic implications, and strategies such as supplementation, food enrichment, and crop enrichment can help mitigate the disease [7].

A deficiency of iron among young children may cause permanent cognitive and developmental disabilities. Motor and social-emotional outcomes improve significantly with early iron supplementation, particularly when supplemented before the manifestation of severe deficiency [8]. Childhood iron deficiency can occur without anemia and affect body tissues. The most common causes are rapid growth, low birth weight, and excessive consumption of cow's milk. Its treatment involves identifying the cause, iron supplementation, and adopting better eating habits [9].

Anemia affects more than 25% of the world's population, and iron deficiency is the primary cause of over half of these cases. Early-life iron deficiency is especially risky in children younger than seven and results in compromised brain development, neurocognitive and neurodevelopmental disorders, and permanent abnormalities of brain regions like the hippocampus and dopaminergic system [10,11].

Even iron deficiency, without clinically evident anemia, has been linked to cognitive deficits in children, specifically in attention, intelligence, and behavior [12]. Further, the mental and behavioral outcomes among iron-deficiency anemic children are not markedly improved with iron therapy. This suggests that early iron deficiency may lead to permanent developmental consequences, which the supplement cannot completely rectify [13]. Iron deficiency is also prevalent among teenage girls and has been linked to decreased intelligence, memory, concentration, and academic performance. These cognitive deficiencies are observed in both anemic and non-anemic individuals [14].

This study will investigate the associations between iron deficiency without anemia and cognitive impairment in children, contributing to a better understanding of the impact of subclinical micronutrient deficiencies on child morbidity and development.

Rationale

Iron deficiency in non-anemic patients has been investigated in most regions of the world, and a range of studies have demonstrated that it can negatively impact children's cognitive abilities, including memory, attention, and learning. These results highlight the importance of low iron levels, even in the absence of anemia, which can still impede a child's mental development. Nevertheless, physical symptoms that are apparent in children with this type of deficiency are usually absent; hence, their condition may go unnoticed and unmanaged during the most crucial developmental stages, which involve growth and learning.

Although previous international research has yielded some significant results, cultural, dietary, and socioeconomic disparities suggest that some of the study findings may not be universally applicable to every population. In Pakistan, where nutritional issues and ignorance concerning hidden deficiencies are prevalent, it would be beneficial to examine the effects of iron deficiency without anemia on the cognitive performance of children in our context. This study examines the association in Pakistani children and aims to fill the gap, as this information can be used to develop more culturally specific health policies and school-based interventions.

Objectives

The study's primary purpose is to investigate the relationship between iron deficiency (without anemia) and cognitive impairment in children. In particular, the research will identify the prevalence of iron deficiency without anemia among children in the chosen Pakistani community and determine the association between the condition and poorer cognitive achievements. The research also clearly assesses iron deficiency without anemia using a self-designed Iron Deficiency Risk Questionnaire (IDRQ), which, while not formally validated, serves as a practical screening tool for the community. The research also examines whether age, gender, dietary habits, socioeconomic status, or other nutritional and demographic factors influence iron levels and mental outcomes. While biochemical confirmation, such as serum ferritin or hemoglobin levels, could provide precise validation, this study employs a community-based screening tool to assess iron deficiency without anemia, acknowledging the limitations of this method. This study aims to provide a clearer understanding of the potential impact of iron deficiency without anemia on child development in the Pakistani context, which will inform early identification and prevention efforts.

## Materials and methods

Study design and methods

The study employed a cross-sectional design to investigate the relationship between iron deficiency without anemia and cognitive impairment in children. The age group of these participants ranged from 5 to under 18 years old. They were recruited at schools and within the community to represent various groups in terms of culture, education level, and socioeconomic status. The methodology enabled a more comprehensive understanding of the potential impact of nutritional factors on cognition in the Pakistani context.

The data were gathered using structured questionnaires that captured information concerning basic demographics, indicators of iron deficiency, and cognitive functioning. The questionnaires were completed by the children themselves or by their parents or guardians, depending on the child's age and understanding. Aside from being informed of the study's purpose, only participants who provided informed consent and assent, along with their guardians, were allowed to participate. The primary purpose of this approach was to determine the possible cognitive effects of iron deficiency without resorting to invasive studies, thereby providing information on a problem often overlooked in child development.

Sample size and technique

The number of children with iron deficiency without anemia in the general population is unknown; therefore, it was considered that the population is infinite in the scope of this study. The size of the sample was determined according to the following formula: 

\[n = \frac{Z^2 \cdot p (1 - p)}{d^2}.\]

In this equation, *Z* denotes the value equal to the desired confidence level, *p* is the estimated proportion considering all the available research, and *d* is the acceptable margin of error. The confidence level was 95%; thus, *Z* was put at 1.96, and *d* was assumed to be 0.05. The sample size was calculated assuming a maximum possible variance (*p* = 0.50), a conservative estimate that ensures the robustness of the study results under variable conditions. This approach is commonly used when there is uncertainty about the proper population proportion, as it provides the maximum sample size required to detect significant effects. Based on this assumption, the minimum required sample size was 385 participants [15]. To account for potential non-responses and incomplete data, we invited a total of 410 participants. After accounting for the expected non-response rate of approximately 6.1%, the final sample size was reduced to 385 participants. This approach ensured that the study maintained sufficient statistical power, even in the event of potential dropouts or incomplete responses.

Sampling of the children was conveniently done in the schools, community centers, and other available areas. Although the sample size was determined using simple random sampling, the sampling technique that was used was convenience sampling. Using this approach enables the researcher to sample participants who fit the inclusion criteria and are reachable and willing to participate. Although this type of convenience sampling can decrease the generalizability of the findings, it was a realistic option based on the context and limited resources of the study. The potential impact of this methodological inconsistency on the generalizability of the results is acknowledged.

The inclusion and exclusion criteria for study participant selection are summarized in Table 1.

**Table 1:** Inclusion and exclusion criteria for study participants.

Inclusion criteria	Exclusion criteria
Children between the ages of 5 and 18 years	Children with a diagnosis of anemia (in the previous medical history, if available)
Being willing to participate in informed consent by parents/guardians	Children who have existing neurological or developmental disorders
Capable of responding to and comprehending questionnaires (or with parental help)	Children receiving iron supplements or treatment for iron deficiency
Able to attend school or accessible at the community level	Failure to respond to a questionnaire appropriately or dropping out during a study

Data collection tools

A standardized questionnaire was administered, which contained three significant parts: demographic details, a screening tool for iron deficiency, and a scale to assess cognitive and behavioral functioning. This combination tool was selected to measure all variables of interest in a single session, thereby reducing participant burden and allowing for extensive data collection.

Demographic information

The initial part obtained general demographical information to investigate the association of age, sex, schooling, household income, and parental education with iron status and cognitive performance. Parents or guardians provided information about the child's overall health status, eating patterns, and some chronic health issues. By recording these background variables, possible confounders were determined, enabling the subgroup analysis of the different socioeconomic classes. 

Table 2 summarizes the demographic items that the parents or guardians responded to. 

**Table 2:** Demographic information.

Items	Responses
Age	-
Gender	-
Educational level of the child	-
Residence	-
Monthly family income	-
Mother’s education level	-
Father’s education level	-
Number of siblings	-
Any known chronic illness in the child?	-
If yes:	-
Any history of blood transfusion in the past 12 months?	-

Iron Deficiency Risk Questionnaire

The second part was a self-created screening tool that would evaluate the probability of having iron deficiency, but not anemia. It contained 11 yes/no questions that focused on the most common signs and dietary habits associated with low iron levels, such as feeling tired, difficulty concentrating, low intake of iron-rich foods, and excessive milk consumption. Each Yes was given 1 point, and the maximum mark was 11. A score of 5 or more was considered indicative of more severe iron deficiency without anemia, based on expert consensus and commonly recognized signs of iron deficiency in children. This threshold was chosen because it aligns with several risk factors associated with iron deficiency in children, such as fatigue, low intake of iron-rich foods, and other common symptoms. However, the IDRQ has not undergone formal validation in a clinical or research setting. It was developed as a preliminary screening tool and not as a diagnostic tool. We acknowledge that future research is needed to validate this tool for wider use formally. Future validation studies will help refine the scoring system and assess the tool’s effectiveness across diverse populations. IDRQ was used in the English language.

It was designed as a convenient, efficient, and non-invasive screening device suitable for both community and school use. Although the IDRQ was not intended as a diagnostic tool, it was created as a convenient community-level screening test to identify children at risk of having non-anemic iron deficiencies due to dietary and behavioral predictors. Further diagnostic testing, such as hemoglobin levels, is recommended for definitive diagnosis, as the IDRQ does not directly assess anemia.

Table 3 displays the 11 items of the self-designed IDRQ.

**Table 3:** Items from the self-designed Iron Deficiency Risk Questionnaire (IDRQ).

Items	Responses
Does the child often feel tired or low on energy without a clear reason?	-
Has the child had difficulty concentrating or paying attention in class?	-
Does the child crave or eat non-food items (e.g., ice, dirt, chalk)?	-
Has the child had a decreased appetite in the past 2-3 months?	-
Have you or others noticed the child looks unusually pale?	-
Has the child had frequent infections or illnesses recently?	-
Does the child eat red meat (beef, mutton) less than twice per week?	-
Does the child drink more than 3 glasses of milk per day?	-
Has the child not been dewormed in the last 6 months?	-
Does the child eat a few iron-rich foods (like spinach, beans, liver)?	-
Has the child recently experienced unexplained hair loss?	-

Strengths and Difficulties Questionnaire (SDQ)

The third section of the questionnaire consisted of a cognitive assessment using the SDQ, developed by Goodman (1997). It has 25 items and five subscales, including emotional symptoms, conduct problems, hyperactivity/inattention, peer relationship problems, and prosocial behavior. The total difficulties score is computed by first assigning each item a 3-point score, with the highest score representing greater difficulties (0 = Not true, 1 = Somewhat true, 2 = Certainly true). The total difficulties score is then the sum of all subscales, except for prosocial behavior. The SDQ has acceptable reliability levels, as indicated by a Cronbach's alpha of approximately 0.73, and is widely applicable in both clinical and community settings. Before SDQ was included in the study, permission to use it was received from the author. In this study, the child or parent/guardian, depending on the child's age and level of understanding, completed the questionnaire [16]. Although the SDQ has been widely validated internationally, including studies in Pakistan that have demonstrated its reliability and applicability to Pakistani populations [17], it has not been specifically validated for the exact population in this study. It is also worth noting that the SDQ was used in its original English version, without translation, for this study. However, its global applicability and successful use in clinical settings in Pakistan further support its utility in our research.

Procedure

The study sample was obtained in Islamabad through schools and community centers, with the informed consent of parents or guardians and the assent of children, as required. Data were collected over five months, specifically between February 2025 and June 2025. The children meeting the inclusion criteria were identified during school hours or community visits. Depending on the age and literacy of the children, the questionnaire was either self-administered by the older children or answered by a trained data collector and a parent. In children between the ages of 5 and 12 years, usually the parent/guardian filled in the questionnaire because younger children did not necessarily possess the mental capacity or literacy level to interpret and answer the questions. In children aged 13 and over, they were asked to answer the questionnaire themselves, with help provided by a parent/ guardian when they had difficulties. Children who found it hard to complete the questionnaire on their own or to understand particular questions were initially identified during the interactions with children on the basis of their level of literacy and understanding, as evaluated at the beginning of the given study. In case the child showed that they were unable to answer the questions, the parent/guardian was assisted with the answers. The information gathered was confidential, and responses were anonymized to ensure the participants' identity was maintained. The study was conducted with an ethical, non-discriminatory, and respectful approach to ensure that the participants represented a diverse range of cultural, educational, and socioeconomic backgrounds, thereby enhancing the quality and representativeness of the study population.

Statistical analysis

Data analysis was carried out using IBM SPSS Statistics 26 (IBM Corp., Armonk, NY). Descriptive statistics, including means, standard deviation, frequencies, and percentages, were used to describe the demographics of the participants. The Kolmogorov-Smirnov and Shapiro-Wilk tests were utilized to determine the normality of data in the IDRQ and the SDQ. A Pearson correlation test was conducted to assess the correlation between scores of IDRQ and SDQ. The independent t-tests were then performed to compare the mean scores of children with and without chronic illnesses on both questionnaires. To analyze the changes in questionnaire scores based on the type of chronic illness and age, a one-way analysis of variance (ANOVA) was applied. To assess the possibility of a significant predictive relationship between IDRQ scores and SDQ scores, a linear regression analysis was carried out. Moreover, chi-square tests were applied to examine the correlations between the categorical age variable, the monthly family income variable, and the type of chronic illness variable. The statistical test procedures were done at a significance level of *P* < 0.05.

Ethical considerations

The research was conducted according to established principles for studying human participants. The research ethics were approved, and an authorization number (IRB-2025-0066) was issued by the Institutional Review Board (IRB) of the NeuroWave Research Center, Islamabad. The research adhered to the concepts of respect, beneficence, and confidentiality. In addition, each participant and their parents or guardians were well-informed about the purpose of the research, the procedures that would be used, and its potential risks and benefits. Consent of parents or guardians was obtained with written informed consent, and where appropriate, assent was taken from children. Participants participated voluntarily and could leave the research without penalty or deprivation of the derived benefit.

The responses were anonymized to uphold confidentiality, and no identifying data was attached to them. During the data-collecting phase, the completeness of responses was verified, and where minor errors were identified, respondents were politely requested to clarify them. Questionnaires with significant missing data that could potentially compromise the quality of the analysis were excluded. This approach ensured data integrity with minimal infringement on the rights and privacy of every participant.

## Results

Table 4 gives the demographic features of the 385 participants. The age description was relatively equal (*n *= 85, 22%) were children aged 8-10 years and (*n *= 83, 22%) were aged 14-16, then there was (*n *= 81, 21%) aged 11-13 and (*n* = 78, 20%) aged 17-18, and (*n* = 58, 15%) aged 5 to 7. The gender distribution was almost balanced, females (*n* = 199, 52%) and males (*n* = 186, 48%). The educational level consisted of middle school (*n* = 104, 27%), primary school (*n* = 101, 26%), high school (*n* = 100, 26%), and preschool (*n* = 80, 21%). The majority of the mothers were educated with primary education (*n* = 110, 29%) and secondary education (*n* = 105, 27%), and were outnumbered by graduates (*n* = 100, 26%) and those without any formal education (*n* = 70, 18%). Similarly, fathers were predominantly those with secondary education (*n* = 110, 29%) and primary education (*n* = 108, 28%), followed by graduates (*n* = 102, 26%), and those with no formal education (*n* = 65, 17%). There was a slightly higher proportion of participants from the rural area (*n* = 213, 55%) than from the urban area (*n* = 172, 45%). The family monthly income was also fairly evenly spread, with the most common income bracket being PKR 51,000 (*n* = 103, 27%), followed by PKR 50,000 (*n* = 102, 27%), and those with incomes less than or more than PKR 25,000 (*n* = 90, 23%). The majority of the children had siblings: three to four siblings (*n *= 115, 30%), one to two siblings (*n* = 110, 29%), greater than four siblings (*n *= 90, 23%), and none (*n* = 70, 18%). Among the children, chronic illness was reported in 207 (54%), with the highest prevalence rates related to asthma (*n* = 36, 18%), thalassemia (*n* = 34, 17%), and epilepsy (*n* = 32, 16%). It is essential to mention that 248 (64%) children had a blood transfusion within 12 months before the collection of the data, with all transfusions being for reasons other than anemia, as children with transfusions due to anemia were excluded from the study. This highlights the high prevalence of severe or chronic health issues within the sample.

**Table 4:** Demographic characteristics of participants (N = 385). All blood transfusions were for reasons other than anemia, as children who received transfusions due to anemia were excluded to focus on iron deficiency without anemia. *f*, frequency, %, percentage

Variable	f	%
Age	-	-
5-7 years	58	15
8-10 years	85	22
11-13 years	81	21
14-16 years	83	22
17≤18 years	78	20
Gender	-	-
Male	186	48
Female	199	52
Child educational level	-	-
Preschool	80	21
Primary school (Grades 1-5)	101	26
Middle school (Grades 6-8)	104	27
High school (Grades 9-12)	100	26
Mother’s educational level	-	-
No formal education	70	18
Primary education	110	29
Secondary education	105	27
Graduate or above	100	26
Father’s educational level	-	-
No formal education	65	17
Primary education	108	28
Secondary education	110	29
Graduate or above	102	26
Residence	-	-
Urban	172	45
Rural	213	55
Monthly family income (PKR)	-	-
<25,000	90	23
25,000-50,000	102	27
51,000-100,000	103	27
>100,000	90	23
Number of siblings	-	-
None	70	18
1-2	110	29
3-4	115	30
>4	90	23
Any chronic illness in the child?	-	-
Yes	207	54
No	178	46
If yes, type of chronic illness	-	-
Asthma	36	18
Thalassemia minor/major	34	17
Epilepsy	32	16
Diabetes	26	13
Celiac disease	22	11
Recurrent infection	25	13
Other	25	12
Any history of blood transfusion in the past 12 months?	-	-
Yes	248	64
No	137	36

Table 5 presents the results of the normality tests for the IDRQ and SDQ, based on the Kolmogorov-Smirnov and Shapiro-Wilk tests. For the IDRQ, the Kolmogorov-Smirnov test yielded a statistic of 0.041 (*P* = 0.200), and the Shapiro-Wilk test produced a statistic of 0.996 (*P *= 0.401). Correspondingly, regarding the SDQ, the value of the Kolmogorov-Smirnov test was 0.035, with a *P*-value of 0.200; the Shapiro-Wilk statistic was 0.998, with a *P*-value of 0.752. In both cases, the *P*-values were greater than 0.05, indicating that the data on both variables were normally distributed. Hence, it was decided that parametric statistical tests could be used in further analysis.

**Table 5:** Normality assessment of study variables using Kolmogorov-Smirnov and Shapiro-Wilk tests (P > 0.05 indicates normal distribution). Data are presented as means ± SD; *P* > 0.05 considered non-parametric; *P* < 0.05 considered significant. df, degree of freedom; SD, standard deviation

Variable	Kolmogorov-Smirnov			Shapiro-Wilk		
	Statistic	df	P	Statistic	df	P
Iron Deficiency Risk Questionnaire	0.041	385	0.200	0.996	385	0.401
Strengths and Difficulties Questionnaire	0.035	385	0.200	0.998	385	0.752

Table 6 indicates a weak positive correlation of 0.272 between the IDRQ and the SDQ. The p-value (< 0.001) suggests that this relationship is statistically significant. Nevertheless, due to the relatively low correlation, there is a weak relationship between the risks of developing iron deficiency and difficulties or strengths identified by the SDQ. This implies that the association is only statistically significant, but the relationship does not represent a high-intensity type of connection that might portray a high magnitude of direct effects.

**Table 6:** Intercorrelation between study variables. **P < 0.001 considered significant; correlation = Pearson correlation.

Variable	Iron Deficiency Risk Questionnaire	Strengths and Difficulties Questionnaire	P
Iron Deficiency Risk Questionnaire	-	0.272	<0.001^**^
Strengths and Difficulties Questionnaire	0.272	-	<0.001^**^

Table 7 compares IDRQ and SDQ scores between children with and without chronic illness. The findings reveal a significant difference in IDRQ scores, with children with chronic illness (*M* = 15.37 ± 1.90) scoring lower than those without chronic illness (*M* = 16.36 ± 2.05); t(385) = -4.893, *P* < 0.001, Cohen’s *d* = -0.50, indicating a medium effect size. Similarly, SDQ scores also differed significantly, with children with chronic illness scoring lower (*M* = 68.52 ± 4.90) than those without chronic illness (*M* = 69.99 ± 5.02); t(385) = -2.915, *P* = 0.004, Cohen’s *d* = -0.30, indicating a small effect size. The study's results demonstrate a strong correlation between chronic disease and the likelihood of being iron-deficient, as well as challenges identified in the behaviors of children.

**Table 7:** Comparison among variables (presence of chronic illness). Data are presented as mean ± SD. Independent t-test; **P < 0.001 considered highly significant. LL, lower limit; UL, upper limit; CI, confidence interval; SD, standard deviation

Variable	Yes (*n* = 207) (*M* ± SD)	No (*n *= 178) (*M* ± SD)	t	P	Cl 95% LL	UL	Cohen’s *d*
Iron Deficiency Risk Questionnaire	15.37 ± 1.90	16.36 ± 2.05	-4.893	<0.001^**^	-1.384	-0.591	-0.50
Strengths and Difficulties Questionnaire	68.52 ± 4.90	69.99 ± 5.02	-2.915	0.004	-2.474	-0.481	-0.30

Table 8 compares the scores of the IDRQ and SDQ in children with varying types of chronic illnesses. The IDRQ revealed a significant difference between the groups, with children who had regular infections (*M* = 17.11, SD = 2.16) and those with celiac disease (*M* = 16.64, SD = 1.68) scoring the highest. A one-way ANOVA reported a significant main effect, *P* < 0.001, and a large effect size (0.145). Similarly, in the SDQ, children with celiac disease scored the highest (*M* = 72.14 ± 6.56), while those with asthma scored the lowest (*M* = 67.15 ± 4.73). An *F*-value of 6.650 and a small effect size (*η*^2^ = 0.096) indicated a significant difference between scores (*P* < 0.001). The findings suggest that the nature of their long-term constitution substantially influences the occurrence of insufficient iron and behavioral problems among children.

**Table 8:** Comparison of variables (type of chronic illness). Data are presented as mean ± SD; *F* = ratio of variance between groups to within groups; η² = effect size; one-way ANOVA; **P < 0.001 considered highly significant. SD, standard deviation; ANOVA, analysis of variance

Variable	Asthma (*n *= 75) (*M* ± SD)	Thalassemia minor/major (*n *= 91) (*M* ± SD)	Epilepsy (*n* = 70); (*M* ± SD)	Diabetes (*n *= 35) (*M* ± SD)	Celiac disease (*n *= 28) (*M* ± SD)	Recurrent infection (*n* = 46) (*M* ± SD)	Other (*n *= 40) (*M* ± SD)	P	*F*(6,378)	*η*2
Iron Deficiency Risk Questionnaire	15.07 ± 1.89	15.25 ± 1.72	15.51 ± 2.05	15.91 ± 2.19	16.64 ± 1.68	17.11 ± 2.16	17.0 ± 1.47	<0.001^**^	10.67	0.145
Strengths and Difficulties Questionnaire	67.15 ± 4.73	68.36 ± 4.00	69.30 ± 4.40	69.14 ± 5.96	72.14 ± 6.56	71.63 ± 4.89	69.98 ± 4.59	<0.001^**^	6.650	0.096

Table 9 presents a comparison of IDRQ scores and SDQ scores across various age groups. For the IDRQ, there was a statistically significant difference in mean scores across age groups (*P* < 0.001), with the lowest score observed in the youngest group (5-7 years; *M* = 14.88 ± 1.55) and the highest in the oldest group (17-18 years; *M* = 16.97 ± 2.02). The *F*-value of 19.118 and *η*² = 0.168 indicate a large effect. Similarly, SDQ scores showed significant differences (*P* < 0.001), with the highest score observed in the 17- to 18-year-old age group (*M* = 72.28 ± 5.97). The *F*-value of 5.837 and *η*² = 0.058 indicate a moderate effect size. These findings highlight the role of age in the risk of iron deficiency and behavioral discomfort in children and teenagers.

**Table 9:** Comparison of variables (age). Data are presented as mean ± SD; *F* = ratio of variance between groups to within groups; *η*² = effect size; one-way ANOVA; **P < 0.001 considered highly significant. ANOVA, analysis of variance; SD, standard deviation

Variable	5-7 years (*n *= 42) (*M* ± SD)	8-10 years (*n *= 132) (*M* ± SD)	11-13 years (*n *= 96) (*M* ± SD)	14-16 years (*n *= 80) (*M* ± SD)	17 ≤ 18 years (*n *= 35) (*M* ± SD)	P	*F*(4,380)	*η*^2^
Iron Deficiency Risk Questionnaire	14.88 ± 1.55	14.98 ± 1.77	16.17 ± 1.92	16.81 ± 2.02	16.97 ± 2.02	<0.001^**^	19.118	0.168
Strengths and Difficulties Questionnaire	68.28 ± 4.08	68.25 ± 4.26	69.02 ± 5.51	70.11 ± 4.91	72.28 ± 5.97	<0.001^**^	5.837	0.058

Table 10 presents a linear regression model predicting SDQ scores based on the IDRQ. A constant value was 58.614 (*P* < 0.001). The SDQ score would be taken when IDRQ is 0. The IDRQ score showed a significant correlation with SDQ scores, with a coefficient of 0.669 (*P* < 0.001), indicating that a one-unit increase in the IDRQ score corresponds to a 0.669-unit increase in the SDQ score. The standardized coefficient (beta) was 0.272, and the relationship between IDRQ and SDQ was moderate and positive. The 95% confidence interval for the IDRQ coefficient was between 0.431 and 0.907, indicating that the actual value is likely to lie within these statistical bounds. These findings demonstrate a significant positive predictive correlation between the risk of iron deficiency and the severity of behavioral difficulties, as assessed using the SDQ.

**Table 10:** Linear regression analysis predicting Strengths and Difficulties Questionnaire (SDQ) scores using the Iron Deficiency Risk Questionnaire (IDRQ). **P < 0.001 considered highly significant. *B*, unstandardized coefficient; SE, standard error; β, standardized coefficient; LL, lower limit; UL, upper limit; CI, confidence interval

Variable	B	95% Cl LL	UL	SE	β	P
Constant	58.614	54.813	62.414	1.933	-	<0.001^**^
Iron Deficiency Risk Questionnaire	0.669	0.431	0.907	0.121	0.272	<0.001^**^

Table 11 presents the distribution of children with various chronic illnesses by age group and monthly family income level, with the highest association observed with both variables (*P* < 0.001). Most of the cases of thalassemia and epilepsy were found among children aged 11-13 and 14-16, whereas the cases of asthma were relatively evenly distributed over young age groups. Older adolescents (14-18 years) had a higher prevalence of celiac disease and other conditions. When it came to income, asthma and thalassemia were found in lower-income families (<50,000 PKR), while celiac disease and other conditions were found in higher-income families (>100,000 PKR). According to the chi-squared tests, chronic disease is a significant factor in the child's age and family income level.

**Table 11:** Descriptive statistics of demographic variables (type of chronic illness, age, monthly family income). *P* = level of significance; *P*-values calculated using the chi-square test. **P < 0.001 considered highly significant. *f*, frequency; df, degrees of freedom; *χ*², effect size

Variables	f	Age (years)					df	P	*X*^2^	Monthly family income (PKR)				df	P	*X*^2^
		5-7	8-10	11-13	14-16	17 ≤ 18				<25,000	25000-50,000	51,000-100,000	>100,000			
Type of chronic illness	-	-	-	-	-	-	24	<0.001^**^	83.4	-	-	-	-	18	<0.001^**^	44.9
Asthma	75	14	34	15	8	4	-	-	-	18	33	17	7	-	-	-
Thalassemia minor/major	91	11	44	21	13	2	-	-	-	18	33	35	5	-	-	-
Epilepsy	70	11	26	17	9	7	-	-	-	20	21	20	9	-	-	-
Diabetes	35	2	14	10	6	3	-	-	-	8	8	15	4	-	-	-
Celiac disease	28	1	3	10	9	5	-	-	-	6	5	6	11	-	-	-
Recurrent infection	46	3	8	12	18	5	-	-	-	7	11	20	8	-	-	-
Other	40	0	3	11	17	9	-	-	-	4	9	20	7	-	-	-

## Discussion

This study investigated the relationship between iron deficiency without anemia and cognitive deficits in Pakistani children aged 5 to 18 years. We observed a modest positive association between iron deficiency risk and cognitive-behavioral problems in children. This finding is consistent with earlier research that has associated iron deficiency, whether or not accompanied by anemia, with deficits in attention, intelligence, emotional control, and behavior. Although the correlation observed in our study is relatively weak, it supports the broader understanding of the cognitive impacts of iron deficiency in the absence of diagnosed anemia [12].

We found that the likelihood of iron deficiency was higher in children with chronic illnesses. This finding is consistent with the existing literature, which suggests that iron deficiency is a common occurrence in chronic diseases and is associated with unfavorable clinical outcomes, underscoring the importance of early screening among these populations [18]. In our study, children with chronic illnesses were at an increased risk of iron deficiency, which may impede cognitive development. This is in line with findings of earlier studies that it is emotional and behavioral challenges in chronically ill children that mediate the adverse effect on mental and academic outcomes [19].

Our study detected a significantly increased risk of iron deficiency in children with celiac disease. This is consistent with current evidence, which suggests that impaired iron absorption in the atypical duodenal mucosa is a common complication of celiac disease, making affected children especially susceptible to the adverse cognitive effects of iron deficiency [20]. In our study, the mental and behavioral difficulties were the highest among children with celiac disease. This has been reinforced by clinical evidence of a temporal relationship between celiac disease and progressive cognitive impairment, which indicates that the condition could contribute to a decline in neurocognitive functioning over a lifespan [21].

Our analyses revealed that the risk of iron deficiency was particularly high among younger children, particularly those between the ages of 5 and 7. This aligns with other evidence indicating that iron deficiency and anemia are more frequent during early childhood, thus necessitating early surveillance and prevention [22]. Our study found that older adolescents experienced more cognitive and behavioral problems than younger adolescents. This correlates with the available literature on how cognitive functions, such as working memory and executive control, decline steadily with age, indicating that cognitive vulnerability can present itself throughout the lifespan [23].

We found that the increased risk of iron deficiency was a significant determinant of cognitive and behavioral problems among children. The present finding aligns with the existing literature on the role of iron deficiency in impairments of attention, intelligence, and emotional regulation, even in the absence of anemia, suggesting that iron plays a significant role in neurodevelopment [12]. Although the regression coefficient (*B* = 0.669) indicates a statistically significant relationship, the effect is relatively small. Nevertheless, a slight change in behavioral difficulty scores can still imply practical significance for academic and developmental performance, notably within vulnerable groups such as children with chronic diseases.

In our research, we found that the nature of chronic ailments varied significantly, depending on the patient's age group, with some diseases being more prevalent during adolescence. This is in line with the previous evidence that indicates multiple chronic conditions have a higher probability of children facing cognitive, behavioral, and physical illnesses, as well as higher utilisation of health care services [24]. We discovered that some chronic diseases were more prevalent in low-income families, pointing to the idea that socioeconomic status plays a role in the distribution of the diseases. This is consistent with the past literature that families with children with chronic conditions experience significant financial burdens and lower standards of living, which can impact access to care and early detection of the illness [25].

Limitations

Although the research contributes valuable intellectuality to the discussion concerning subclinical nutritional deficiencies among children, it is necessary to note its numerous limitations. To begin with, the cross-sectional design cannot be used to make any causal inference. Even though observable relationships were computed, the direction of the relationship between iron deficiency and cognitive impairment cannot be established conclusively. Second, biochemical laboratory testing, such as serum ferritin levels or haemoglobin levels, did not confirm the iron status. Instead, a screening questionnaire was used, which may not be a precise diagnostic method, although it is feasible in the community. Additionally, the IDRQ has not been formally validated, limiting its psychometric reliability and generalizability across different populations. Another weakness is that the data are collected through self-reporting and parent-reporting, which is likely to introduce reporting bias, especially in socioeconomically diverse environments where awareness of food consumption or behavioral guidelines may differ. In addition, SDQ, although extensively validated, provides a generalized picture of cognitive and behavioral difficulties as opposed to neuropsychological profiles. Another limitation pertains to the inclusion of children who had received blood transfusions within 12 months before data collection, even though all transfusions were for reasons other than anemia. This introduces potential confounding effects that were not adequately addressed in the study. Lastly, convenience sampling and unequal urban-rural representativeness can limit or hinder the applicability of findings to broader areas of Pakistan.

Future directions

Future studies should consider using longitudinal studies to understand the causal association and the impact of early treatment for iron deficiency that is not anemic on long-term cognitive outcomes. Adding biochemical verification of iron status will increase the validity of the results and allow for comparisons between the levels of severity of the deficiency. More specific cognitive domains (e.g., executive function, working memory) should also be considered when administering neuropsychological and behavioral scales, such as the SDQ. Feedback to rural and underserved populations, where the research needs to be extended, with specific attention to regional dietary and cultural differences, will provide more detailed information for policymaking. Nutrition education school-based programs, such as routine iron screening, should also be piloted and assessed regarding their effectiveness in addressing cognitive and academic outcomes across all subjects.

## Conclusions

The results of this study underscore a significant association between nonanemic iron deficiency and cognitive-behavioral complications in Pakistani children. The low iron level was also correlated with higher scores on the SDQ scale, even when there was no clinically visible anemia, indicating that subclinical iron deficiency can affect a child's brain and mental development, as well as their adaptation skills. The findings raise concern about the need for early identification, community awareness, and nutritional programs in schools and communities to avoid the silent scourge of iron deficiency. It is essential to solve these problems in advance to improve children's developmental progress and the quality of their lives.
